# Global health policy and neglected tropical diseases: Then, now, and in the years to come

**DOI:** 10.1371/journal.pntd.0005759

**Published:** 2017-09-14

**Authors:** Thomas Fürst, Paola Salari, Laura Monzón Llamas, Peter Steinmann, Christopher Fitzpatrick, Fabrizio Tediosi

**Affiliations:** 1 Department of Epidemiology and Public Health, Swiss Tropical and Public Health Institute, Basel, Switzerland; 2 University of Basel, Basel, Switzerland; 3 School of Public Health, Imperial College London, London, United Kingdom; 4 Independent consultant, Basel, Switzerland; 5 Swiss Centre for International Health, Swiss Tropical and Public Health Institute, Basel, Switzerland; 6 Department of Control of Neglected Tropical Diseases, World Health Organization, Geneva, Switzerland; University of Washington, UNITED STATES

In the year 2000, the eight Millennium Development Goals (MDGs) were adopted by the United Nations and its member states [1]. Even though health aspects that mostly affect the poor were explicitly included in the MDGs (goals 4, 5, and 6), many stakeholders felt that important conditions were not adequately represented or were unjustly omitted [1, 2]. Among the anonymous “other diseases” mentioned in MDG 6 was a cluster of diseases that became known as neglected tropical diseases (NTDs). Their omission was a call to arms for the NTD stakeholders and a stimulus to join forces to gain critical mass. Key arguments were the considerable geographical overlap of NTDs, the commonness of co-infections related to shared risk factors, their poverty-driven and poverty-promoting characteristics, and potential synergies in fighting them. The little resources devoted to NTDs in relation to their burden and the availability of comparatively cheap options for treatment and prevention further encouraged the coordination of specific efforts to address the NTDs and to develop the NTD brand [3, 4].

In 2003, the Foundation for Innovative Diagnostics (FIND; see www.finddx.org) and the Drugs for Neglected Diseases Initiative (DNDi; see www.dndi.org) were set up. Two years later, the World Health Organization’s (WHO) NTD department was established. In 2006, the United States Agency for International Development (USAID) launched its NTD programme. In 2007, the scientific journal *PLoS Neglected Tropical Diseases* published its first issue. Also in 2007, the WHO’s *Global Plan to Combat NTDs 2008–2015* was finalised [5], which started to consolidate previous efforts to design integrated helminthiasis control approaches [6] and thoughts about intensified intersectoral and interprogrammatic collaboration [7]. The *Global Plan* grouped the NTDs into “tool-ready” diseases (i.e., those with inexpensive and easily scalable strategies, such as preventive chemotherapy, available) and “tool-deficient” diseases (i.e., those with only comparatively costly and difficult-to-manage tools, such as early detection and treatment, as the best option) [5]. The efforts to provide an integrated framework to control and prevent NTDs were further advanced in the *First WHO Report on NTDs* (2010), which recommended 5 combinable key strategies: (i) preventive chemotherapy; (ii) intensified case management; (iii) vector control; (iv) safe water, sanitation, and hygiene (WASH); and (v) veterinary public health [8]. This *First WHO Report* also provided a list of World Health Assembly (WHA) resolutions pertaining to NTDs and an analysis of how they contribute to attaining the MDGs (for an updated list of NTD-related WHA resolutions, see http://www.who.int/neglected_diseases/mediacentre/resolutions/en/) [8]. In 2012, WHO published *A Roadmap for Implementation* with the aim to accelerate the control and elimination of NTDs by setting clear, disease-specific targets [9]. Inspired by WHO’s *Roadmap*, the *London Declaration on NTDs* was written and endorsed by a number of public and private organisations, institutions, and pharmaceutical companies that formed the Uniting to Combat NTDs coalition in the same year [10]. The coalition committed to support the eradication of dracunculiasis, the elimination of lymphatic filariasis, leprosy, human African trypanosomiasis, and blinding trachoma, and the control of schistosomiasis, soil-transmitted helminthiasis, chagas, visceral leishmaniasis, and onchocerciasis by 2020 [10]. In recognition of the progress and to urge continued efforts, the key strategies and targets were enshrined in the first ever WHA resolution (66.12) on all NTDs in 2013 [11]. *Uniting to Combat NTDs* started to produce annual scorecards to provide status updates [10], and WHO published its *Second* (2013), *Third* (2015), and—very recently—*Fourth Report on NTDs* (2017) [12–14].

During this journey, public–private partnership approaches proved successful in developing innovative tools against NTDs and in helping to secure unprecedented resources. Main bilateral donors such as USAID and the Department for International Development (DfID) of the United Kingdom, international organisations such as WHO and the World Bank, philanthropic foundations such as the Bill & Melinda Gates Foundation, nongovernmental development organisations, academia, the endemic countries themselves, and pharmaceutical companies were critical in gaining and sustaining momentum. For instance, the support of the latter started in 1987 with ivermectin donations from Merck & Co., Inc., for onchocerciasis control, gained increasing traction over the years, and now comprises donations of 14 different drugs from 10 companies with an estimated worth of US$2–$3 billion annually [2, 5, 8–13].

In September 2015, the Sustainable Development Goals (SDGs) were adopted as a successor of the MDGs until 2030. The NTDs are now explicitly mentioned under target 3.3 of the health-related SDG3, and progress is measured against the specific indicator 3.3.5, “Number of people requiring interventions against NTDs” [15]. With this highest policy level breakthrough and the successful public–private partnership approach, which has also been branded as a “rags-to-riches story” by WHO Director-General Margaret Chan [16], what else needs to be done in terms of health policy?

It is important to note that being considered in the vast SDGs agenda will not automatically ensure or even further raise the profile of NTDs. Furthermore, MDG-related structures, longer-term commitments, and connections will not automatically be adjusted to the SDG goals. As with the MDGs, the cross-cutting linkages of NTDs with various SDGs should be continuously stressed and cross-sectoral and transdisciplinary collaborations sought. Besides the well-known, mutually beneficial links—for instance, with efforts to “end poverty in all its forms everywhere” (SDG1) or with strains to “ensure availability and sustainable management of water and sanitation for all” (SDG6)—new allies may emerge [17]. One potentially very powerful tie from within the health sector could be with the paradigm of universal health coverage (UHC; SDG target 3.8) [14, 15]. UHC is likely to be one of the leading health policy paradigms in the years to come and is defined by the 3 dimensions that (i) all people in need (ii) can use promotive, preventive, curative, rehabilitative, and palliative health services of sufficient quality (iii) without suffering from financial hardship [18]. Evidently, UHC can only be achieved if people affected by or at risk of NTD infections also receive appropriate health services. Hence, UHC offers a strong leverage to insist on interventions against NTDs to all in need, and at the same time, as NTDs are often most prevalent in the most neglected and poorest populations, the NTDs can serve as a “litmus test” for UHC [13, 14].

Unfortunately, so far, UHC and NTD policies have been developed separately and are often disconnected. Little guidance is available on which specific NTD-related activities within the 5 key strategies should be prioritized for inclusion in UHC benefit packages. For instance, most strikingly, the key strategy “intensified case management” may include a large variety of activities—potentially ranging from simple antibacterial treatment to surgery—and to social inclusion programmes, depending also on the country-specific endemicity of the different NTDs. Concerns have been raised that UHC policies could lead to an increasing focus on health facility-based care rather than prevention, giving lower priority to more community-based treatment and often also cross-sectoral, NTD-relevant interventions such as vector control, veterinary public health, and WASH, even though the latter activities are also of greatest benefit to the least well off. This currently still unclear situation is further complicated by the fact that the current effective coverage of various NTD-relevant health services is insufficiently documented in many endemic countries.

As another challenge, the *Third WHO Report on NTDs* estimated investment targets for universal coverage against NTDs and concluded that a large part of funding is unlikely to come from foreign donors [13]. At the same time, simple combination of estimates about people requiring interventions against NTDs with estimates about people living below the severe poverty threshold suggests that, overall, between 54% and 89% of those requiring NTD interventions in the 21 countries with the highest NTD burdens live in severe poverty (Table 1). Apparently, country-specific differences exist, but a considerable number of people requiring interventions against NTDs will be unable to substantially contribute to financing these interventions. Consequently, the UHC paradigm will necessitate (and should also be harnessed for) the redesigning of policies for the collection, pooling, and reallocation of investments and revenues in endemic countries and to identify additional inland financing strategies for NTD interventions over the next years.

**Table 1:** Populations requiring interventions against neglected tropical diseases (NTDs) and living in poverty. To predict the percentage and the total number of people requiring interventions against NTDs and living below US$3.10 purchasing power parity (PPP) per day and person, 2 extreme scenarios were considered. The “optimistic” scenario assumes that the percentage of people living in severe poverty is the same among those who do and those who do not require NTD interventions. However, the proportion of people living in severe poverty is likely to be higher in those requiring NTD interventions than the national average as NTDs are usually most prevalent among the poor. The “pessimistic” scenario assumes that those who require NTD interventions are first of all those who live in severe poverty.

Country	Total population estimates, 2014^a^	Total number of people requiring NTD interventions, 2014^b^	Percentage of total population requiring interventions against NTDs, 2014	Percentage of total population living below US$3.10 PPP per day^c^	Percentage of people requiring interventions against NTDs living below US$3.10 PPP per day		Total number of people requiring interventions against NTDs living below US$3.10 PPP per day	
					Optimistic scenario	Pessimistic scenario	Optimistic scenario	Pessimistic scenario
China	1,368,930,561	26,227,888	2	11	11	100	2,885,068	26,227,888
India	1,294,737,310	577,240,673	45	58	58	100	334,799,590	577,240,673
Indonesia	254,308,847	127,979,175	50	36	36	72	46,072,503	92,145,006
Brazil	205,973,640	18,680,873	9	8	8	88	1,494,470	16,439,168
Pakistan	185,021,056	47,386,262	26	37	37	100	17,532,917	47,386,262
Nigeria	177,463,719	140,381,164	79	77	77	97	108,093,496	136,169,729
Bangladesh	159,086,957	49,873,889	31	57	57	100	28,428,117	49,873,889
Philippines	99,132,703	44,803,112	45	38	38	84	17,025,183	37,634,614
Ethiopia	96,938,457	67,843,988	70	71	71	100	48,169,231	67,843,988
Democratic Republic of the Congo	74,892,895	57,568,918	77	91	91	100	52,387,715	57,568,918
Myanmar	53,458,639	40,777,860	76	NA	NA	NA	NA	NA
Tanzania	51,832,105	33,868,257	65	76	76	100	25,739,875	33,868,257
Sudan: North	39,384,299	28,468,689	72	39	39	NA	NA	NA
Sudan: South				64	64	NA	NA	NA
Uganda	37,796,069	25,344,345	67	65	65	97	16,473,824	24,584,015
Nepal	28,181,459	21,352,583	76	48	48	63	10,249,240	13,452,127
Mozambique	27,215,953	22,815,820	84	88	88	100	20,077,922	22,815,820
Ghana	26,770,192	18,697,745	70	49	49	70	9,161,895	13,088,422
Madagascar	23,577,196	20,491,358	87	91	91	100	18,647,136	20,491,358
Cameroon	22,772,412	19,449,659	85	44	44	52	8,557,850	10,113,823
Mali	17,090,697	19,462,713	ND (>100%)	78	78	NA	NA	NA

^a^ Data on country-specific total numbers of population and annual rates of population change are from the United Nations Population Prospects (see https://esa.un.org/unpd/wpp/). Total population estimates for 2015 and average annual rates of population change from 2010 to 2015 were used to calculate total population estimates for 2014.

^b^ Data on country-specific total number of people requiring interventions against NTDs in 2014 are from the World Health Organization Global Health Observatory (see http://apps.who.int/gho/data/node.sdg.3-3-map-5?lang=en).

^c^ Data on country-specific proportions of people living in severe poverty are from the World Bank Open Data Database (see http://data.worldbank.org/indicator/). To define severe poverty, the recently updated international poverty line of US$3.10 purchasing power parity per day and person was applied.

NA, not available; ND, not determined; PPP, purchasing power parity.

Obviously, much has been achieved in the fight against NTDs over the past years with regards to (global) health policy and financing, but major challenges are still ahead (Box 1). In order to address these challenges and sustain the momentum also when NTD prevalences and morbidities dwindle, it may be advisable to complement the original rallying cry about the “neglect” of the respective tropical diseases. Major achievements and particularly historic opportunities for success in the control, elimination, and—in the cases of dracunculiasis and yaws—even eradication of NTDs should be strongly emphasised to ensure a continued buy-in from existing partners and to mobilise additional stakeholders.

**Box 1. Highlights.**

- The omission of the NTDs in the MDGs was a call to arms for the respective stakeholders and a stimulus to join forces to gain critical mass.
- Since then, the NTD brand has been forcefully developed, and advocacy led to the establishment of new organisations and collaborations and recognition by national and international institutions.
- Public–private partnerships have proven highly successful in securing unprecedented resources and also developing tools and strategies to support the fight against NTDs.
- In the SDGs, the NTDs are explicitly mentioned under target 3.3 (“By 2030, end the epidemics of AIDS, tuberculosis, malaria, and neglected tropical diseases, and combat hepatitis, water-borne diseases, and other communicable diseases”).
- To sustainably support and strengthen the fight against NTDs, opportunities for cross-cutting linkages and cross-sectoral and transdisciplinary collaborations in the SDG agenda should be actively sought.
- The paradigm of UHC (SDG target 3.8) should become a natural ally—the UHC movement can help to highlight the still substantial coverage and investment gaps in the fight against NTDs, and the NTDs can serve as a “litmus test” for UHC.
- Historic opportunities for success in the fight against NTDs should be strongly emphasised in the years to come and complement the original branding about the neglect of the respective tropical diseases—this may help to ensure the continued support by old and new partners and also when NTD prevalences and morbidities dwindle.
